# Effects of extrinsic rearfoot posting in custom foot orthoses on frontal plane kinematics and kinetics

**DOI:** 10.1186/1757-1146-5-S1-O8

**Published:** 2012-04-10

**Authors:** Scott Telfer, Mandy Abbot, Daniel Rafferty, Jim Woodburn

**Affiliations:** 1 School of Health and Life Sciences, Glasgow Caledonian University, Glasgow, UK

## Background

A regularly prescribed design variable in foot orthoses (FOs) is the addition of an extrinsic rearfoot post, a feature which can be angled medially or laterally and is intended to control movement of the calcaneus during the stance phase of gait [1]. This study aims to investigate whether introducing incremental changes in this feature will produce a linear trend in the user’s frontal plane biomechanical responses, and whether responses vary between normal and pronated feet.

## Materials and methods

Ten participants were recruited: five healthy controls and five patients with a symptomatic pronated foot type. Computer aided design (CAD) models of a pair of customised FOs were produced from a 3D surface scan of the subject’s feet using orthotic design software. These devices were manufactured and checked for comfort and fit. The original CAD design was subsequently altered to produce nine additional FO designs (for one randomly chosen foot) with posting levels varying in 2° steps from 6° lateral to 10° medial and these were then manufactured. After wearing the original FOs for one week, participants came to the gait laboratory for assessment and kinematic and kinetic measurements of the lower limbs were made during gait for each orthotic condition.

## Results

**Figure 1:**
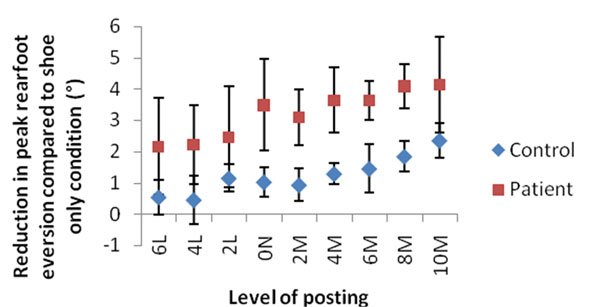
Reduction in peak rearfoot eversion for control and patient groups across all orthotic conditions. 6L: 6° lateral post; 0N: neutral posting; 10M: 10° medial post. Error bars are ±1SD.

Linear trends for the reduction of peak rearfoot eversion were measured (control group R^2^=0.9, P=0.003; patient group R^2^=0.86, P=0.04) across the tested orthotic conditions (Figure 1). Differences in the effects of the devices on peak rearfoot eversion in the control and patient group were found to be significant (P<0.001). Changes in ankle and knee adduction moments were not significant for trends or between groups.

## Conclusions

These results provide preliminary quantitative mode of action evidence for the prescription of personalised FOs intended to control rearfoot eversion through the use of an extrinsic rearfoot post. Care should be taken when extrapolating results from FO research carried out on normal foot types to clinical populations.
